# A simple and rapid technique to achieving an airtight seal for negative pressure wound therapy in externally fixated lower limb open fractures

**DOI:** 10.1308/rcsann.2023.0062

**Published:** 2023-11-20

**Authors:** L Boyce, C Jordan, G Pafitanis

**Affiliations:** ^1^ Barts Health NHS Trust, London, UK; ^2^ University of Cyprus, Nicosia, Cyprus

## Background

External fixation and negative pressure wound therapy (NPWT) dressings are routinely used in the management of lower limb open fractures.^1^ Achieving an airtight seal for NPWT can be challenging when multiple pins are inserted close to the debrided wound. Once negative pressure is applied, tearing of the adhesive film on the fixation hardware results in loss of the vacuum seal.^2^

**Figure 1:**
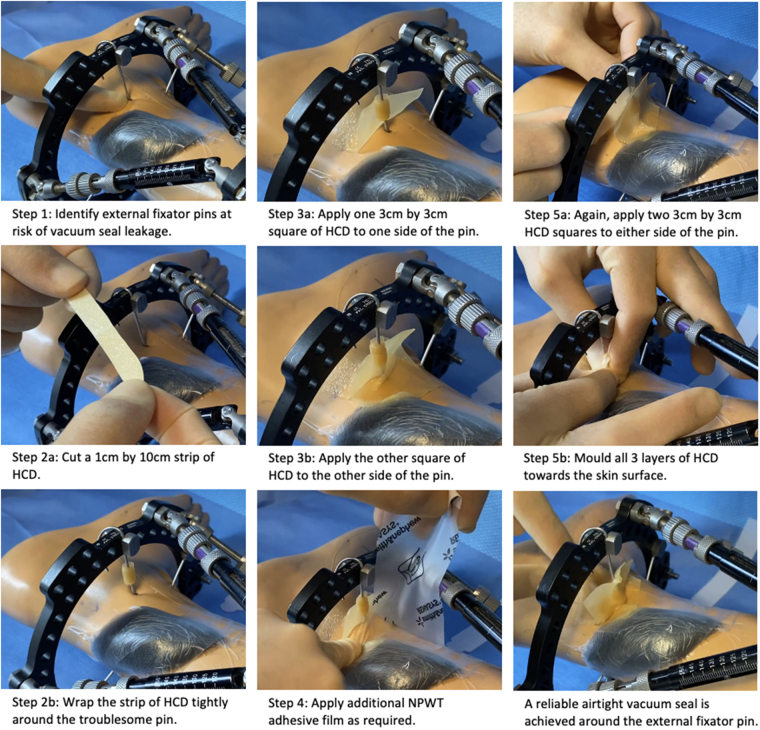
The simple five-step technique to achieving an airtight vacuum seal for negative pressure wound therapy in externally fixated open fractures

We developed a five-step technique that exploits the elastic, adhesive and mouldable properties of hydrocolloid dressings (HCDs) to enhance the vacuum seal around external fixator pins (Figure 1).

## Technique

Our technique had the following steps:

•  Step 1: identify external fixator pins at risk of vacuum seal leakage – pins entering the skin within 2cm of the wound edge are candidates for this technique. Apply the NPWT foam and adhesive film to the wound.
•  Step 2: cut a 1 × 10cm strip of HCD and wrap it tightly around the troublesome pin.
•  Step 3: cut two identical 3 × 3cm squares of HCD and apply them to either side of the pin, overlying the adhesive film on the skin surface and sandwiching the HCD strip wrapped around the pin.
•  Step 4: apply additional adhesive film as required, overlying the HCD already applied.
•  Step 5: apply two further identical 3 × 3cm squares of HCD either side of pin and film. Mould all three layers of HCD towards the skin surface.

## Discussion

Alternative solutions to this problem have previously been described, despite numerous flaws.^3–5^ HCDs are cheap, sterile, durable and easy to remove. Our technique provides a standardised method for ensuring an optimal, time-efficient vacuum seal using simple HCD.
